# Systematic Review: AI Applications in Liver Imaging with a Focus on Segmentation and Detection

**DOI:** 10.3390/life15020258

**Published:** 2025-02-08

**Authors:** Mihai Dan Pomohaci, Mugur Cristian Grasu, Alexandru-Ştefan Băicoianu-Nițescu, Robert Mihai Enache, Ioana Gabriela Lupescu

**Affiliations:** 1 Department 8: Radiology, Discipline of Radiology, Medical Imaging and Interventional Radiology I, University of Medicine and Pharmacy “Carol Davila”, 050474 Bucharest, Romania; mihai-dan.pomohaci@drd.umfcd.ro (M.D.P.); alexandru-stefan.baicoianu@drd.umfcd.ro (A.-Ș.B.-N.); 2 Department of Radiology and Medical Imaging, Fundeni Clinical Institute, 022328 Bucharest, Romania; robert-mihai.enache@rez.umfcd.ro

**Keywords:** liver, hepatocellular carcinoma, cholangiocarcinoma, artificial intelligence, machine learning, deep learning, CT, MRI, US

## Abstract

The liver is a frequent focus in radiology due to its diverse pathology, and artificial intelligence (AI) could improve diagnosis and management. This systematic review aimed to assess and categorize research studies on AI applications in liver radiology from 2018 to 2024, classifying them according to areas of interest (AOIs), AI task and imaging modality used. We excluded reviews and non-liver and non-radiology studies. Using the PRISMA guidelines, we identified 6680 articles from the PubMed/Medline, Scopus and Web of Science databases; 1232 were found to be eligible. A further analysis of a subgroup of 329 studies focused on detection and/or segmentation tasks was performed. Liver lesions were the main AOI and CT was the most popular modality, while classification was the predominant AI task. Most detection and/or segmentation studies (48.02%) used only public datasets, and 27.65% used only one public dataset. Code sharing was practiced by 10.94% of these articles. This review highlights the predominance of classification tasks, especially applied to liver lesion imaging, most often using CT imaging. Detection and/or segmentation tasks relied mostly on public datasets, while external testing and code sharing were lacking. Future research should explore multi-task models and improve dataset availability to enhance AI’s clinical impact in liver imaging.

## 1. Introduction

The liver is the largest organ in the abdomen, normally positioned in the upper-right quadrant, acting as a biofilter, with multiple metabolic tasks including both exocrine and endocrine functions [1]. Its unique dual blood supply both from the hepatic artery and the portal vein reflects its complex role in maintaining homeostasis; the hepatic veins collect blood from the liver and deliver it to the inferior vena cava [2]. The impact of chronic liver disease, cirrhosis and its complications are extensive, with a need for better prevention and surveillance methods [3]. This need is further emphasized by the increasing prevalence of metabolic dysfunction-associated fatty liver disease (MAFLD), estimated to grow by 21% from 2015 to 2030 [4]. Metabolic dysfunction-associated steatohepatitis (MASH) is part of MAFLD and is characterized by fat accumulation, inflammation and fibrosis, often progressing to cirrhosis [5]. Advanced liver imaging provides a non-invasive assessment of these changes, reducing the need for procedures like biopsy. Transient elastography and shear wave elastography (SWE) can evaluate liver stiffness, aiding in the staging of fibrosis [6]. Magnetic resonance elastography (MRE) has emerged as a highly accurate modality for detecting fibrosis, with improved reproducibility over ultrasound-based methods [7]. Steatosis can also be diagnosed with ultrasound (US) imaging but has no precise method of non-invasive quantification. With the advent of MRI proton density fat fraction (PDFF), a more precise quantification of hepatic steatosis can be performed [8].

Primary liver cancer frequently develops in the setting of chronic liver disease, represented mainly by hepatocellular carcinoma (HCC) but also by cholangiocellular carcinoma and other rare entities [9]. In 2020, the Global Cancer Observatory classified primary hepatic cancer as the third most common cause of death, ranking it as the sixth most frequently diagnosed type of cancer [9]. The liver is also a common site of metastasis, with up to 50% of patients presenting with liver metastasis or developing them during their oncologic disease, particularly from colorectal and pancreatic cancer [10]. Computed tomography (CT) and magnetic resonance imaging (MRI) are crucial in diagnosing and monitoring these patients. Similarly, contrast-enhanced ultrasound (CEUS) is a key technique that can provide additional real-time assessment of liver lesions.

**Figure 1:**
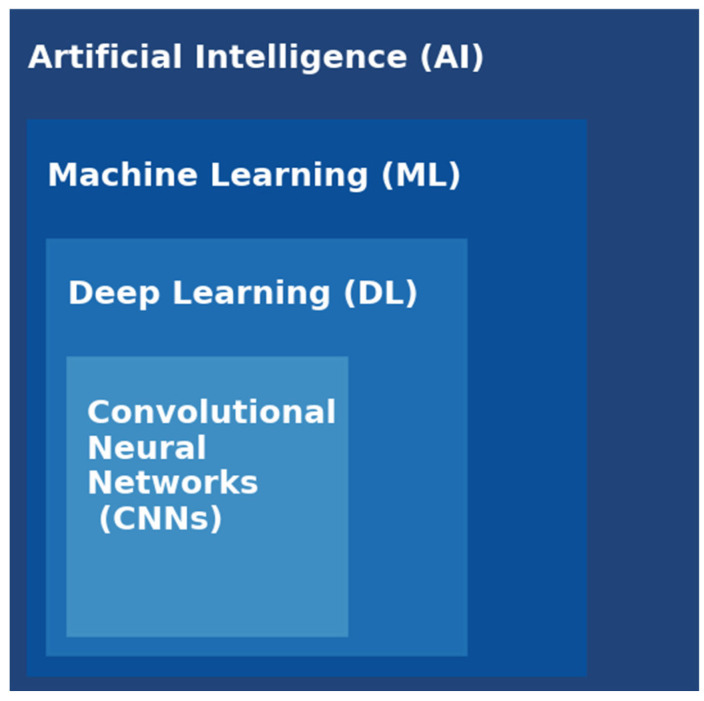
Simplified hierarchical representation of AI subcategories.

Artificial intelligence (AI) is a growing field of study in the context of an increase in the amount of available data and computational power. Machine learning (ML) and deep learning (DL) are two nested subfamilies of AI, capable of extracting data without explicit programming [11]. Convolutional neural networks (CNNs) are a type of DL inspired by the function of neurons and synapses in the human cortex that can extract patterns of features from images during the training phase and use them to give an output during the testing phase [12]. CNNs, compared to other ML subtypes, do not require hand-crafted features or manual segmentation, so minimal human intervention is required. However, they demand large amounts of data and advanced graphical processing units [12]. A simplified hierarchical representation of the relationship between these AI subcategories is represented in Figure 1. Radiology and diagnostic imaging are a major areas of research for DL and ML applications [13,14], as the data are stored in a picture archiving and communication system (PACS) for multiple years and can be retrospectively processed. Even though the number of commercially available AI applications in radiology is increasing, the abdominal region is lagging behind in the implementation of these technologies. One meta-analysis of 100 commercially available applications from 2020 showed that only 2% focused on the liver, compared to 38% on neuro-imaging and 31% on chest. Additionally, these two applications were specifically designed for iron and fat quantification. In an analysis of the trends of applications for DL networks in medical imaging [15], the abdominal region ranked third between 2012 and 2020, behind neuro- and thoracic imaging. Some potential explanations for this paucity in liver applications are the complexity of triple-phase contrast scans with arterial, porto-venous and delayed/equilibrium phases, adding the difficulty of registration. Additionally, the liver is more prone to changes in orientation or artifacts secondary to respiratory movement and diaphragmatic excursions.

To ensure reproducibility and transparency, guidelines for medical imaging AI model development have been published, such as Checklist for Artificial Intelligence in Medical Imaging (CLAIM) [16] or MINimum Information for Medical AI Reporting (MINIMAR) [17]. A comprehensive list of guidelines for developing AI tools has been outlined by Klontzas et al. [18]. Code sharing plays a crucial role in the reproducibility and validation of AI models in medical imaging. This allows researchers to verify, refine and build upon existing algorithms, fostering collaboration and accelerating innovation. Similarly, prospective studies are essential for the correct validation of AI models.

The main AI tasks in radiology are detection, segmentation, classification/regression and image optimization/reconstruction tasks. A detection model identifies a structure, an organ or a lesion, most often using a bounding box. DL models, especially CNNs, can be used to identify liver lesions, assisting radiologists and potentially reducing the number of overlooked lesions [19]. Segmentation models can create a precise delineation of the pixels representing a structure in an image [20], outputting a mask. They can help automate processes like CT or MRI liver volume assessment for transplant patients, fat quantification in MAFLD patients using MRI-PDFF and the evaluation of fibrotic changes in patients with chronic liver disease using MRE. Similarly, lesions can be precisely delineated, providing 3D measurements for diagnosis and follow-up. A classification task categorizes an image into a variable number of categories [20] (e.g., hepatocarcinoma or cholangiocarcinoma or hemangioma, etc.), while a regression task will use an image or a set of images to output a continuous value [21] (e.g., predicted survival = 1.2 years). All these models can improve the daily workflow of radiologists, providing precise, reproducible measurements and novel biomarkers in liver imaging.

The rationale for this systematic review lies in the increasing interest in AI-based applications for liver imaging in radiology in the context of a growing global burden of liver diseases and the parallel advancements in AI technologies. The objective of this review was to systematically assess and classify research studies on AI applications in liver radiology from 2018 to 2024. Specifically, our research questions were the following: Considering the complexity of hepatic imaging, what are the main anatomical areas of interest (AOIs) in liver studies developing AI models? Considering the great potential and multitude of AI tasks in liver imaging, what is the prevalence of classification, detection, segmentation and image optimization models in the evaluated studies? What imaging modalities are most frequently used in liver AI research? How has the distribution of AOI, AI task and modality changed over time (2018–2024)? A more detailed analysis was performed on detection and segmentation studies, with the following questions: What are the most common AOIs specifically for this task? What percentage of studies rely on public, private, or a combination of both datasets? What are the most commonly used public datasets? To what extent is external validation applied in liver detection and segmentation studies? What percentage of studies provide publicly available code and how does the lack of code sharing impact reproducibility and transparency? Are AI-based liver imaging studies predominantly retrospective or prospective?

## 2. Materials and Methods

This study adhered to the Preferred Reporting Items for Systematic Reviews and Meta-Analyses (PRISMA) guidelines [22].

A systematic search was performed on the PubMed/Medline, Scopus and Web of Science databases, including articles published between 01.01.2018 and 29.10.2024. The following keywords were used in combination with BOOLEANS operators according to each database’s specific search queries: “Liver”, “Hepatic”, “Liver Metastasis”, “Hepatocarcinoma”, “Cholangiocarcinoma”, “Radiology”, “Diagnostic Imaging”, “Magnetic Resonance Imaging”, “MRI”, “computed tomography”, “CT”, “Ultrasonography”, “Ultrasound”, “Artificial Intelligence”, “Machine Learning”, “Deep Learning”, “Radiomics” and “neural network”. Detailed information on the search queries for each database is provided in the Supplementary Data (Table S1).

**Figure 2:**
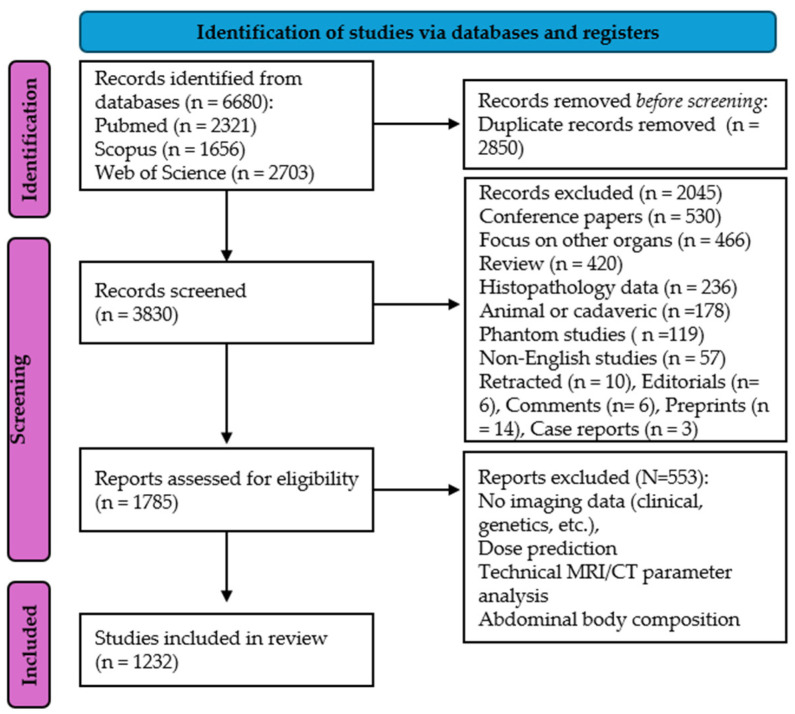
Prisma flow chart.

The extracted records included 6680 articles, which were uploaded to the Rayyan application (https://www.rayyan.ai/): PubMed—2321; Scopus—1656; and Web of Science—2703. Using the dedicated functionality, duplicates were detected and removed, leaving 3830 articles for the screening phase. Two radiologists (R.E., 3rd-year resident; M.P., radiologist) independently screened the data using Rayyan labels and titles. Inclusion criteria: original research articles, publication dates of 1 January 2018–29 October 2024, English language, papers applying to humans, the use of AI and radiological images (CT, MRI, US, PET-CT, etc.) and focusing on the liver. A total of 2045 articles were removed based on the following exclusion criteria: conference papers (530), a focus on other organs (466), reviews (420), histopathology data (236), animal or cadaveric experiments (178), phantom studies (119), non-English studies (57), retracted publications (10), preprints (14), editorials (6), comments (6) and case reports (3). For the remaining 1785 articles, the titles and abstracts were analyzed by three radiologist reviewers (R.E., M.P., A.S.B.N), and 553 articles were excluded for the following reasons: they were studies with no imaging data (radiology reports, lab data, genetics, etc.), dose predictions, a technical MRI/CT component analysis or abdominal body composition studies. There were 379 conflicts, which were resolved by consensus agreement. The Prisma flow diagram of our work is represented in Figure 2.

The remaining 1232 articles were classified according to the following:

- The area of interest (AOI): liver parenchyma, lesions, vascularity, bile ducts or complex models (applied to >1 area, e.g., liver parenchyma and lesions).
- The AI task: detection and/or segmentation, classification/regression, image optimization (registration/synthesis/reconstruction) and multi-task (performing >1 task, e.g., segmentation and classification).
- The modality: US, CT, MRI, nuclear medicine, multi-modality (using > 1 modality, e.g., CT and MRI).

**Figure 3:**
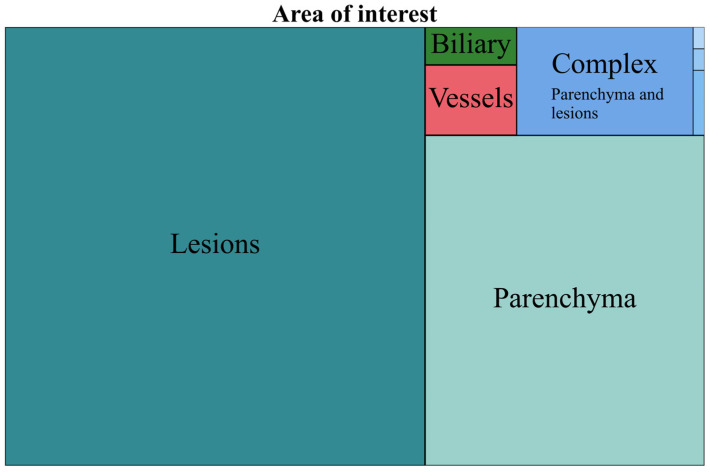
Tree chart representing AOIs.

**Figure 4:**
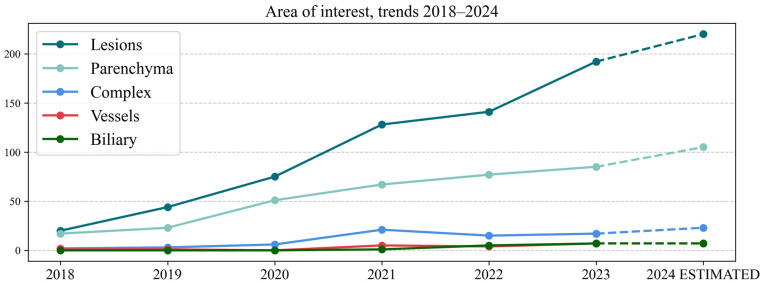
Line chart representing AOIs in 2018–2024.

**Figure 5:**
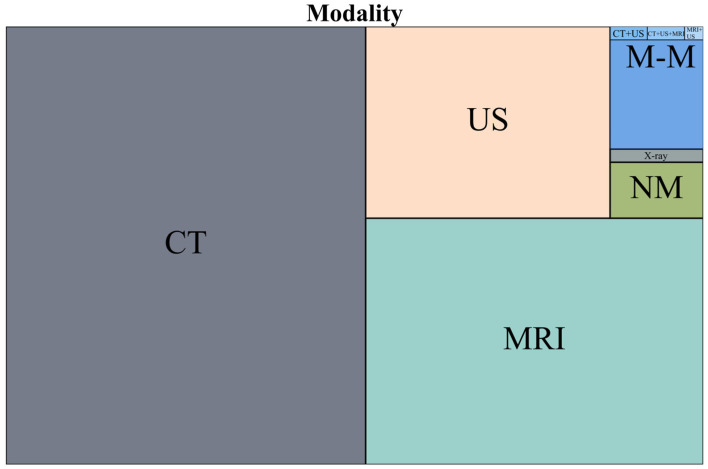
Tree map representing modality (M-M = multi-modality, NM = nuclear medicine).

**Figure 6:**
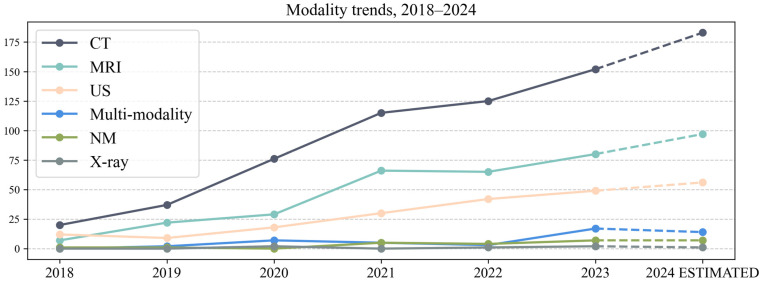
Line chart representing modality use, 2018–2024.

**Figure 7:**
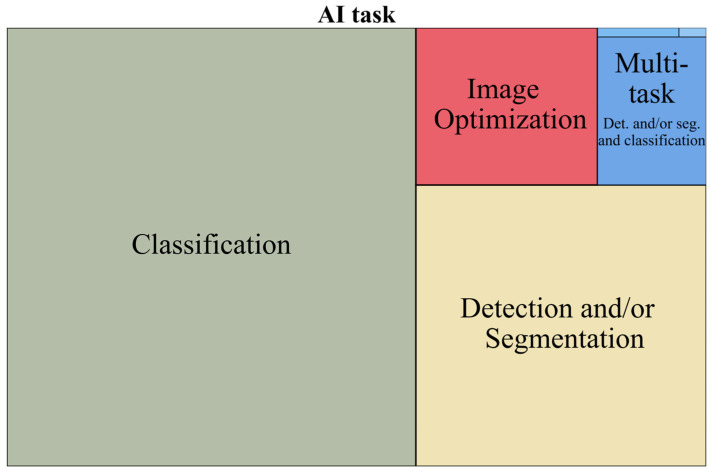
Tree map representing AI tasks.

**Figure 8:**
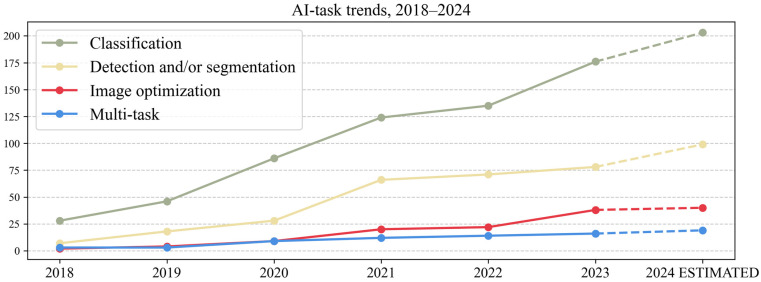
Line chart representing AI tasks, 2018–2024.

**Figure 9:**
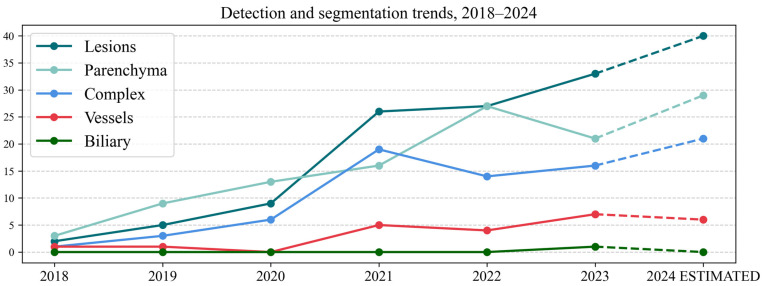
Line chart representing detection and/or segmentation AI tasks, 2018–2024.

**Figure A1:**
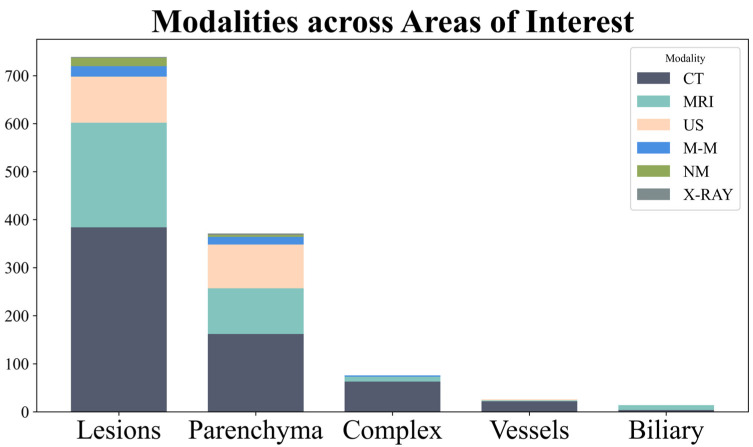
Stacked bar chart representing modalities across AOIs.

**Figure A2:**
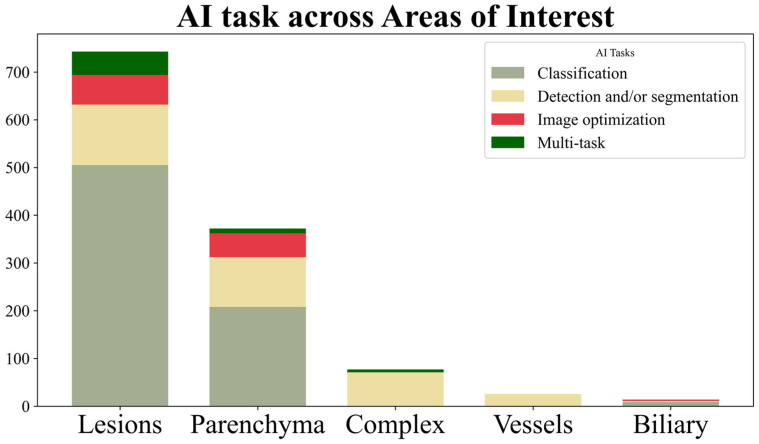
Stacked bar chart representing AI tasks across AOIs.

**Figure A3:**
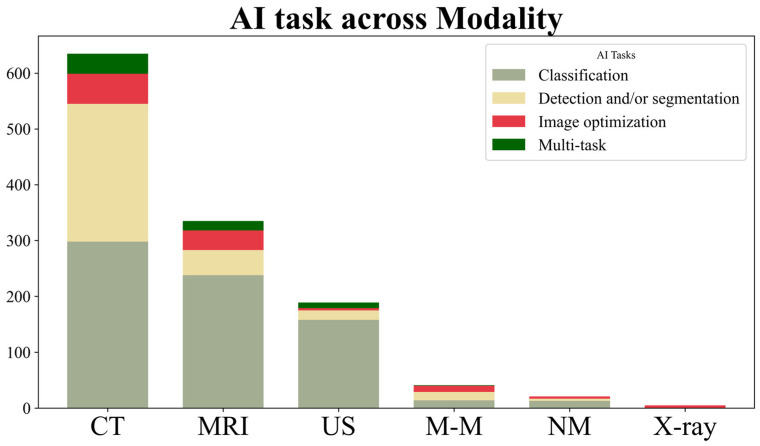
Stacked bar chart representing AI tasks across modality.

We performed a more in-depth analysis of the full manuscripts for articles that focused on detection and/or segmentation (329/1232), extracting information on the following:

- The datasets used: public, private or both.
- The usage of an external dataset.
- The availability of the code used for model development.
- The prospective or retrospective nature of the study.
- Data from individual studies were tabulated according to the abovementioned criteria to summarize key study characteristics. Visual representations included hierarchical tree maps (Figures 3, 5 and 7) for the AOI, modality and AI task; line charts (Figures 4, 6, 8 and 9) for temporal trends; and stacked bar charts in Appendix A (Figure A1, Figure A2 and Figure A3). The number of articles for which the respective data was unavailable is mentioned in the results.

## 3. Results

### 3.1. Area of Interest (AOI)

Data regarding the AOI are represented in Table 1, with all the five main categories in the left section and a subgroup analysis of complex AOIs in the right section. No. represents the number of articles that researched that AOI. The tree chart in Figure 3 helps visualize the relatonship between the five main areas of interest in a hierarchical configuration. The yearly trends of the same five main AOIs from 2018 to 2024 are represented in Figure 4 (the values for 2024 have been estimated).

### 3.2. Modality

The results on the modality used are found in Table 2, with the main six types in the left section and a more detailed analysis of multi-modality subgroups in the right section. No. represents the number of articles that used that modality. The tree chart in Figure 5 was generated to help visualize the hierarchy between the six main categories. Figure A1 in Appendix A is a stacked bar chart representing the modalities across the AOIs. The yearly trends from 2018 to 2024 are shown in Figure 6 (the values for 2024 have been estimated). For six articles, information on the modality used was not found; these were not added to the table or figures.

### 3.3. AI Task

Data on the main four AI tasks is represented in Table 3 in the left section, with a further analysis of AI multi-task subcategories in the right section. No. represents the number of articles that applied the AI task. A visual representation of the hierarchical structure of the main four AI tasks is represented in Figure 7. Stacked bar charts representing the relationship of AI task across AOI and AI task across modality are shown in Figure A2 and Figure A3 in Appendix A. The yearly trends from 2018 to 2024 are represented in Figure 8 (the values for 2024 have been estimated).

### 3.4. Detection and/or Segmentation Studies

#### 3.4.1. Detection and/or Segmentation AOIs

An in-depth analysis of the 329 articles that researched detection and/or segmentation tasks was performed. The main AOIs studied in these papers are represented in Table 4 in the left section, with a subcategory analysis of complex AOIs in the right section. The yearly trends from 2018 to 2024 are represented in Figure 9 (the values for 2024 have been estimated).

#### 3.4.2. Detection and/or Segmentation (D&S) Datasets

The distribution of public and private datasets is shown in the left section of Table 5, with the top four public datasets used represented in the right section. No. represents the number of articles that used that particular dataset or dataset type. For 17 articles, information regarding datasets was not found; these were not added to the tables. A list of all the public datasets found in the evaluated papers can be found in Appendix A, Table A1.

## 4. Discussion

Several reviews have previously analyzed the role of AI in liver imaging, systematically or by summarizing the state of the art. Nam et al. investigated publications n hepatology with a broader area of research, including studies using radiology, histopathology and clinical data [27]. Their analysis also suggested that CT was the most widely used modality and that diagnosis and prognosis were the most common functions, followed by segmentation. Unlike Nam et al., who emphasized the potential of AI in different data types, our review focused on radiology data only and had a systematic approach. Furthermore, we categorized research based on imaging modalities, AI tasks, and the areas of interest, with a detailed focus on detection and segmentation database usage and code-sharing practices. Radiya et al. systematically analyzed 191 studies and focused specifically on machine learning applications in CT imaging. Our review expanded this scope by incorporating other types of radiology data (MRI, US, multi-modality, nuclear medicine) and providing temporal trends from 2018 to 2024 [28]. Their study also analyzed dataset types, with public being the most common and LITS the most widely used. Additionally, we provided a list of all the public datasets that were found in detection and segmentation studies and performed an analysis of the data described in private datasets.

In our systematic review, liver lesions were the most researched AOI, explored in 60.30% of studies. This superiority was maintained across the years (Figure 4) and across imaging modalities (Figure A1) and AI tasks (Figure A2). The number of articles that handled complex AOIs was low (6.25%) but showed a slow and steady increase, shown in Figure 4 (a peak in 2021 with 21 studies). The majority of complex AOI studies (93.50%) combined an analysis of liver parenchyma and lesions. There was only one article that handled >2 AOIs, a model developed by Oh et al. that segmented parenchyma, lesions, vessels and bile ducts in MRI hepatobiliary phase [29]. Furthermore, when performing a cross analysis of complex AOI articles and AI tasks, we noticed that most of them (92.20%) involved detection and/or segmentation (Figure A2). These data might suggest that having a comprehensive approach and integrating all its structures into one AI model is still a very difficult task.

CT was the primary imaging modality, used in 51.42% of papers, almost twice more often than MRI (27.19%) and three times more often than US (15.34%). This preference can be explained by multiple factors. CT uses Hounsfield Unit (HU) to measure voxel values, which provides a standardized and reproducible measure across different scanners. In contrast, MRI lacks a similar quantitative standard, and the signal intensity can vary depending on scanner and imaging protocols. This variability introduces an additional preprocessing step to normalize the data, an essential step in developing MRI AI models, which lacks uniform guidelines [30]. MRI public datasets are also lacking; we found only six in the analyzed D&S group of articles, namely CHAOS [26], AMOS22 [31], ATLAS [32], DLDS [33], LiverHccSeg [34] and TCIA [35]. Ultrasound imaging is more user-dependent and the windows used to capture liver images are not standardized. Another factor could be the scarcity of public US datasets; in our D&S group of articles, we only found one, MICCAI CLUST [36,37]. The list of all the public datasets found in the evaluated papers is present in Appendix A, Table A1.

Analyzing the trends for modality use (Figure 6), we can see an increase in using multi-modality data from 5 studies in 2021 and 3 studies in 2022 to 17 in 2023. One explanation could be the popularity of foundation models, which increased in 2023, which stimulated an interest in combining data from multiple modalities for advanced analysis and applications. In addressing the need for collaborative frameworks and improved dataset diversity, the CHAIMELEON project focuses on developing a standardized, multi-modal imaging repository for AI tool validation across Europe. Similarly, the European Federation for Cancer Images (EUCAIM) project wishes to establish a federated infrastructure for secure, cross-border data sharing. By leveraging these frameworks, future research can bridge the gap between AI development and clinical application, ultimately enhancing AI’s impact in liver imaging and beyond.

Classification and/or regression were the most researched tasks in our study on liver imaging. The purpose of such models can range from distinguishing benign vs. malignant lesions [38,39] to more specific differential diagnoses like HCC vs. combined cholangiocarcinomahepatocarcinoma [40,41]. They can also be developed to subtype a tumor according to histopathological (HP) features, like predicting microvascular invasion in HCC [42,43] or predicting response to treatment in cholangiocarcinoma [44,45] or survival in HCC [46,47]. The multitude of options that this task can encompass could also be an explanation for these superior numbers, beyond just an increased interest.

The distribution of AI tasks across the modalitiy highlights key differences in research focus and modality preferences (depicted in Figure A3 in Appendix A). In publications that used CT, classification tasks represented 46.92% of CT studies, while detection and segmentation (D&S) represented 38.89% of CT studies. The CT D&S studies followed the same pattern of AOI distribution as other modalities, most focusing on liver lesion segmentation, with liver parenchyma segmentation as the second most common AOI. CT classification studies had diverse objectives, with most focusing on liver parenchyma (e.g., fibrosis staging [48], NASH diagnosis [49], etc.) or liver lesions (e.g., prediction of HCC microvascular invasion [50]). Conversely, in publications that used MRI, classification tasks prevailed, representing 71.04% of MRI studies, with detection and segmentation accounting for 13.43% of MRI studies. The MRI classification studies were also very diverse in purpose, most focusing on liver parenchyma (e.g., fibrosis evaluation on MRI ADC maps [51]) or liver lesions (e.g., predicting HCC recurrence after ablation [52]). This distribution suggests that CT is widely used for both lesion characterization and segmentation, while MRI plays an essential role mainly in AI lesion characterization or AI-based predictions and less in detection and segmentation. This can be explained by the superior complexity of MRI liver imaging, used often as an additional diagnostic tool when US and CT imaging cannot provide a diagnosis. The superior contrast resolution and multiple types of acquisition can allow for more information to be extracted by AI models in order to reach a complex diagnosis like microvascular invasion in HCC [43], which normally requires a histopathological diagnosis.

With the increasing need to extract more complex imaging biomarkers, there is also a need for automated processes that output at least a rudimentary delineation of the area of interest, if not precise 3D volumes. Manual segmentations, although considered a “gold standard”, are prone to inter- and intra-reader variability and are also time-consuming [53]. In a study conducted on 105 patients, implementing an automatic DL model reduced the processing time for liver segmentation from an average of 169.8 s per case for manual contouring to 1.7 s [54]. This was our motivation to perform a more in-depth analysis of detection and/or segmentation studies.

An analysis of the trends for segmentation and detection (Figure 9) showed that in 2018–2020, the number of articles focusing on liver parenchyma was greater than those focusing on lesions. After 2021, the ratio reversed or equalized (2022). One possible explanation is that liver parenchyma segmentation had very good results in LiTS competitions in 2017 and 2018 [23], with most of the teams obtaining Dice scores higher than 0.920. Another factor could be that many studies now focus on multi-organ segmentation, with competitions like Medical Segmentation Decathlon (MSD) [55] assessing the segmentation performance for 10 organs in total. More recently, Total Segmentator has been publicly released, which provides segmentations for 104 structures [56], including the liver, and it has been implemented in open-source applications like 3Dslicer 5.0 (https://www.slicer.org).

The good performance of DL models for liver parenchyma segmentation is also reflected in studies analyzing clinical impact, with graft volume estimations performed by DL models closely matching the actual graft weight found using both CT [57,58,59,60,61] and MRI [62]. Radiomics features extracted from automated hepatic parenchyma segmentations have also been shown to be more reproducible compared to manual contours in portal-phase MRI [63]. This data could suggest that AI liver parenchyma segmentation might be a solved problem from a technical performance perspective and should be ready for clinical implementation.

Studies have shown that a unidimensional diameter does not always correlate with the actual tumoral size and volume [64,65,66], and there is an increase in the inter-reader variability [67]. In a study by Joskowicz et al. [68], when radiologists had access to quantitative AI data for liver metastasis evaluation, they changed and improved their oncological disease status decision in 1/3 of cases. Similarly, in a study by Wesdorp et al. [69], total tumor volume quantification for colorectal liver metastasis demonstrated prognostic potential in response evaluation to systemic treatments compared to unidimensional measurements. These studies underline the need for AI-assisted quantifications in liver oncologic studies and the need to move beyond unidimensional measurements. Results from LiTS competitions [23] for liver lesion detection and segmentation showed a maximum Dice score of 0.702 in 2017 and 0.739 in 2018, and the best detection performance was 0.479 in 2017 and 0.554 in 2018. Although these competitions provide a common set of rules for participation, which ensures transparency, there is less information on patient history or multiphase scan integration. Liver imaging is very complex and clinical data is essential, reflected in the 81 defined terms for image interpretation in the Liver Imaging Reporting and Data System (LI-RADS) Lexicon [70].

A closer look into segmentation and detection (D&S) articles showed that LiTS [23] (used in 41.64% of studies) and 3DIRCADb [24] (used in 29.48%) were the most popular public datasets, used alone or in combination with other datasets. More than 1/4 of D&S studies (27.65%) used only one public dataset for model development, and only 10% of articles explicitly mentioned using an external dataset for model testing. Current guidelines like CLAIM [16] or MINIMAR [17] emphasize the presence of external data for model development in medical imaging. The most common combination of two datasets was LiTS [23] and 3DIRCAD [24], used in 35 studies (10.63%). When combining these datasets, it is important to keep in mind that 3DIRCADb is already contained within LiTS and to avoid using it as a test set, which will falsely improve the performance.

We found 155 studies that used private datasets (alone or combined with public ones). For 52.25%, information on contrast acquisition or the MRI sequence was not found. In papers that described their data, most private datasets (29.67%) used complex data, including multiphasic CT/MRI, multiple non-contrast MRI acquisitions, or a combination of both. A complete description of the imaging data found in private datasets is presented in Appendix A in Table A2. Using multiphasic imaging is strongly recommended for liver tumor imaging in clinical practice, especially if the etiology is unknown [71]. Using more than one phase for DL segmentation has been shown to improve accuracy and reduce the number of false negative predictions for hepatocarcinoma [72]. However, this adds complexity, especially with liver registration between acquisitions, which has been shown to be a source of false positives or false negatives for DL models [73]. Understanding the context where these AI models perform best or worse with regard to data type could help us better integrate these models into clinical practice.

Most detection and/or segmentation studies (74.77%) were described as retrospective, while only were prospective (0.91%); the rest (23.31%) of the studies had no information on the retro- or prospective nature of collecting data. Studies using only public datasets were considered retrospective. Prospective studies are essential for AI model development, as they allow for real-time validation in clinical settings, reducing the risk of dataset bias and overfitting. Code sharing is recommended and mentioned as a checklist item in the CLAIM guidelines [16]; it ensures reproducibility, transparency and collaboration for AI model development. A multi-society statement by the ACR, ESR and other leading radiological organizations emphasized the ethical responsibility of AI developers to promote openness and equitable access to AI tools [74]. These ethical principles align with the need for code sharing, as it allows for critical scrutiny, validation and continuous improvement by the global research community. Despite being included as a strong recommendation or as a mandatory part of scientific articles in most journals, sharing practices in medical sciences remain low [75]. In our study, code links were shared by a small number of D&S papers, 36/329 (10.94%); the full list of these articles is provided in the Supplementary Materials in Table S2 [54,59,63,73,76,77,78,79,80,81,82,83,84,85,86,87,88,89,90,91,92,93,94,95,96,97,98,99,100,101,102,103,104,105,106,107].

As we understand the importance of classification and/or regression models in literature we plan to make a similar in-depth analysis of these types of studies in the future.

## 5. Limitations

By combining detection and segmentation in our AI task assessment, we might have lost some of the granularity in liver task evaluation. However, these terms are sometimes used interchangeably, and even when both are mentioned, sometimes metrics are present for only one of the tasks. Our decision to group them together was made to maintain consistency in data reporting. However, future studies could benefit from distinguishing these tasks more clearly and incorporating standardized evaluation metrics to improve comparability across studies.

The number of articles regarding biliary imaging might have been underrepresented as no specific keywords for biliary structures were included in the search. This limitation could impact our findings by underestimating the role of AI in evaluating biliary pathologies. Future research could refine keyword selection to include terms related explicitly to the biliary system, ensuring a more comprehensive review of AI applications in this area. Similarly, image synthesis, reconstruction or reproducibility, represented in our study by the “Image quality” group, might also not be well represented, since these models are frequently applied to multiple regions of the body and the ones focused on liver do not reflect the global impact of these types of studies. This limitation suggests that our findings may not capture the full scope of AI-driven image optimization methods. Future studies could consider a more extensive review of multi-organ AI applications and their liver-specific implications.

Our data for 2024 was incomplete (collected up to 10/2024); therefore, in all the trends figures, to avoid a false downward trend of the slope, we used a linear regression to estimate the values for 2024. While this approach provided a reasonable estimation, it introduced uncertainty into the 2024 projections. Future studies incorporating complete data from 2024 will be necessary to validate our trend observations.

## 6. Conclusions

This systematic review highlights the main areas of research for liver AI applications. Liver lesions emerged as the primary area of interest (60.30%), while complex models addressing multiple liver structures remain scarce. CT was the most widely used imaging modality (51.54%), benefiting from greater dataset availability, while MRI and ultrasound faced challenges due to variability and limited datasets. CT was widely used for both classification and segmentation studies, while MRI was mostly used for classification tasks. For detection and/or segmentation studies, public datasets such as LiTS and the 3DIRCADb were most popular for AI model development. However, their limited diversity and the low use of external testing (10%) can pose difficulties with generalizability. Most studies were retrospective (74.77%), with minimal code sharing (10.94%), a factor that might reduce the reproducibility and clinical adoption. Complex models that integrate multiple AOIs and tasks are still lacking. Future research should prioritize the development of diverse datasets, robust external validation and prospective studies to bridge existing gaps. Greater transparency through open-access code sharing and adherence to reporting guidelines will further support the integration of AI into clinical practice.

## Figures and Tables

**Table 1:** AOIs and its subcategory complex AOI numbers of articles.

AOI	No.	% of Total	Complex AOI	No.	% of Complex	% of Total
Lesions	743	60.30	Parenchyma and lesion	72	93.50	5.84
Parenchyma	372	30.19	Parenchyma and vessels	3	3.89	0.24
Complex	77	6.25	Lesions and vessels	1	1.29	0.08
Vessels	26	2.11	Parenchyma, lesion, vessels and biliary	1	1.29	0.08
Biliary	14	1.13				

**Table 2:** Modality and its subcategory multi-modality numbers of articles.

AOI	No.	% of Total	Multi-Modality	No.	% of Complex	% of Total
CT	635	51.54	CT and MRI	36	87.80	2.92
MRI	335	27.19	CT and US	2	4.87	0.16
US	189	15.34	CT and US and MRI	2	4.87	0.16
Multi-Modality	41	3.32	MRI and US	1	2.43	0.08
Nuclear Medicine	21	1.70				
X-Ray	5	0.40				

**Table 3:** AI tasks and its subcategory AI multi-task numbers of articles.

AI Task	No.	% of Total	AI Multi-Task	No.	% of Complex	% of Total
Classification	723	58.68	Detection and/or segmentation and classification	61	93.84	4.95
Detection and/or segmentation	329	26.70	Detection and classification	3	4.61	0.24
Image optimization	115	9.33	Detection, segmentation and classification	1	1.53	0.08
Multi-task	65	5.27				

**Table 4:** Detection and/or segmentation AOIs and its subcategory complex AOI numbers of articles.

Segmentation and/or Detection AOI	No.	% of Complex	% of Total	Detection and/or Segmentation Complex AOI	No.	% of Complex	% of Total
Lesions	128	38.90	10.38	Parenchyma and lesions	66	92.95	5.35
Parenchyma	104	31.61	8.44	Parenchyma and vessels	3	4.22	0.24
Complex	71	21.58	5.76	Parenchyma, lesions, vessels and biliary	1	1.40	0.08
Vessels	25	7.59	2.02	Lesions and vessels	1	1.40	0.08
Biliary	1	0.30	0.08				

**Table 5:** Segmentation and/or detection dataset type and most commonly found public datasets.

Dataset Type	No.	% of D&S Studies	Main Public Datasets	No.	% of D&S Studies
Public	158	48.02	LiTS [23]	137	41.64
Private	99	30.09	3DIRCADb [24]	97	29.48
Public and private	56	17.02	SLIVER07 [25]	25	7.59
			CHAOS [26]	23	6.99

## Data Availability

The data extracted from the included studies can be provided on reasonable request.

## Supplementary Materials

The following supporting information can be downloaded at https://www.mdpi.com/article/10.3390/life15020258/s1: Table S1: Literature search strategy; Table S2: Articles with public script.

## Abbreviations

The following abbreviations are used in this manuscript:

PRISMA	Preferred Reporting Items for Systematic Reviews and Meta-Analyses
CT	Computed tomography
MRI	Magnetic resonance imaging
US	Ultrasound
NALFD	Non-alcoholic fatty liver disease
HCC	Hepatocarcinoma
AI	Artificial intelligence
ML	Machine learning
DL	Deep learning
CNN	Convolutional neural network
PACS	Picture archiving and communication system
CLAIM	Checklist for Artificial Intelligence in Medical Imaging
MINIMAR	MINimum Information for Medical AI Reporting
AOI	Area of interest
NM	Nuclear medicine
M-M	Multi-modality
LiTS	Liver Tumor Segmentation
3DIRCAD	3D image reconstruction for comparison of algorithm database
SLIVER	Segmentation of the Liver
CHAOS	Challenge-combined healthy abdominal organ segmentation
MSD	Medical Segmentation Decathlon
CLUST	Challenge on Liver Ultrasound Tracking
AMOS	Abdominal multi-organ segmentation
ATLAS	A Tumor and Liver Automatic Segmentation
DLDS	Duke liver dataset
ACT-1K	Abdomen-CT1k
BTCV	Beyond the cranial vault
NM	Nuclear medicine
M-M	Multi-modality

## Appendix A

**Table A1:** List of public datasets found in the detection and/or segmentation articles.

Dataset	Used by No. of Articles	Modality
Liver Tumor Segmentation (LiTS) [23]	137	CT
3D image reconstruction for comparison of algorithm database (3DIRCAD) [24]	97	CT
Segmentation of the Liver (SLIVER) [25]	25	CT
Challenge-combined healthy abdominal organ segmentation (CHAOS) [26]	23	CT and MRI
Medical Segmentation Decathlon (MSD) [55]	13	CT
The cancer imaging archive (TCIA) [35]	4	CT and MRI
Challenge on Liver Ultrasound Tracking (CLUST) [36,37]	3	US
Abdominal multi-organ segmentation (AMOS) [31]	2	CT and MRI
A Tumor and Liver Automatic Segmentation (ATLAS) [32]	2	MRI
Duke liver dataset (DLDS) [33]	2	MRI
LIVERHCCSEG [34]	2	MRI
Abdomen-CT1k (ACT-1K) [108]	2	CT
ISICDM [109]	1	CT
Beyond the cranial vault (BTCV) [110]	1	CT
KAGGLE zxcv2022 [111]	1	CT
Multi-organ Abdominal CT Reference Standard Segmentations [112]	1	CT
VISCERALAnatomy [113]	1	CT

**Table A2:** List of main acquisition phases and MRI sequences used in the detection and/or segmentation papers using private datasets.

Dataset Type Used	Used by No. of Articles
No information	81 (52.25%)
Multiphasic CT/MRI	33 (21.29%)
Single-phase CT/MRI—venous	13 (8.38%)
Multiphase multiparametric MRI	8 (5.16%)
Single-phase MRI—hepatobiliary	6 (3.87%)
Multiparametric MRI (non-contrast)	5 (3.22%)
**Single-sequence MRI-T1 (non-contrast)**	3 (1.93%)
Single-phase CT/MRI—arterial	2 (1.29%)
Single-phase CT/MRI—delayed	2 (1.29%)
Single-sequence MRI-T2	1 (0.64%)
Single-sequence MRI-PDFF	1 (0.64%)
