# Supplementary material for: Systematic Review: AI Applications in Liver Imaging with a Focus on Segmentation and Detection
Source: Life (Basel). 2025 Feb 8;15(2):258. doi: 10.3390/life15020258 (PMC11856300; doi:10.3390/life15020258)
Supplement: Supplementary file 1 [file life-15-00258-s001.zip › life-3426837-supplementary.pdf]

Table S1. Literature search strategy

Search number		Query
Pubmed		(("Liver"[MeSH] OR "liver"[Title/Abstract] OR "hepatic"[Title/Abstract] OR "Liver Neoplasms"[MeSH] OR "Carcinoma, Hepatocellular"[MeSH] OR "hepatocarcinoma"[Title/Abstract] OR "Cholangiocarcinoma"[MeSH] OR "cholangiocarcinoma"[Title/Abstract]) AND ("Radiology"[MeSH] OR "radiology"[Title/Abstract] OR "Diagnostic Imaging"[MeSH] OR "imaging"[Title/Abstract] OR "Magnetic Resonance Imaging"[MeSH] OR "MRI"[Title/Abstract] OR "magnetic resonance imaging"[Title/Abstract] OR "Tomography, X-Ray Computed"[MeSH] OR "computed tomography"[Title/Abstract] OR "CT"[Title/Abstract] OR "Ultrasonography"[MeSH] OR "ultrasound"[Title/Abstract]) AND ("Artificial Intelligence"[MeSH] OR "artificial intelligence"[Title/Abstract] OR "Machine Learning"[MeSH] OR "machine learning"[Title/Abstract] OR "Deep Learning"[Title/Abstract] OR "Radiomics"[Title/Abstract] OR "Neural Networks, Computer"[MeSH] OR "neural network"[Title/Abstract]) OR "transformers"[Title/Abstract])) AND2019 to 2024 (last date 29th of October)
Scopus		(TITLE-ABS-KEY ("liver")OR TITLE-ABS-KEY ("hepatic")OR TITLE-ABS-KEY ("hepatocarcinoma")OR TITLE-ABS-KEY ("cholangiocarcinoma")) AND (TITLE-ABS-KEY ("radiology")OR TITLE-ABS-KEY ("imaging")OR TITLE-ABS-KEY ("MRI")OR TITLE-ABS-KEY ("magnetic resonance imaging")OR TITLE-ABS-KEY ("computed tomography")OR TITLE-ABS-KEY ("CT")OR TITLE-ABS-KEY ("ultrasound")) AND (TITLE-ABS-KEY ("artificial intelligence")OR TITLE-ABS-KEY ("machine learning")OR TITLE-ABS-KEY ("deep learning")OR TITLE-ABS-KEY ("radiomics")OR TITLE-ABS KEY ("neural network")) AND PUBYEAR > 2019 AND PUBYEAR < 2024 (last date 29th of October)
WEB OF SCIENCE	1#	(TI=(liver) OR TI=(hepatic) OR TI=(HEPATOCARCINOMA) OR TI=(CHOLANGIOCARCINOMA)) AND (TI=(radiology) OR TI=(imaging) OR TI=(MRI) OR TI=(MAGNETIC RESONANCE IMAGING) OR TI=(COMPUTED TOMOGRAPHY) OR TI=(CT) OR TI=(ultrasound)) AND (TI=(artificial intelligence) OR TI=(machine learning) OR TI=(deep learning) OR TI=(radiomics) OR TI=(NEURAL NETWORK))
	2#	(AB=(liver) OR AB=(hepatic) OR AB=(HEPATOCARCINOMA) OR AB=(CHOLANGIOCARCINOMA)) AND (AB=(radiology) OR AB=(imaging) OR AB=(MRI) OR AB=(MAGNETIC RESONANCE IMAGING) OR AB=(COMPUTED TOMOGRAPHY) OR AB=(CT) OR AB=(ultrasound)) AND (AB=(artificial intelligence) OR AB=(machine learning) OR AB=(deep learning) OR AB=(radiomics) OR AB=(NEURAL NETWORK))
	3#	(AK=(liver) OR AK=(hepatic) OR AK=(HEPATOCARCINOMA) OR AK=(CHOLANGIOCARCINOMA)) AND (AK=(radiology) OR AK=(imaging) OR AK=(MRI) OR AK=(MAGNETIC RESONANCE IMAGING) OR AK=(COMPUTED TOMOGRAPHY) OR AK=(CT) OR AK=(ultrasound)) AND (AK=(artificial intelligence) OR AK=(machine learning) OR AK=(deep learning) OR AK=(radiomics) OR AK=(NEURAL NETWORK))

http://doi.org/10.3390/life15020258

Table S2. Articles with public script ([41,46,50,61–93])

https://github.com/mjirik/imcut	10.1088/1361-6560/aabd19	Liver segmentation
https://github.com/RanSuLab/RAUNet-tumor-segmentation.git	https://doi.org/10.3389/fbioe.2020.605132	Liver lesion segmentation
https://github.com/licongsheng/DCSegNet	10.1109/ACCESS.2020.3012990	Liver segmentation
https://github.com/liver-research/LiverCAD.	https://doi.org/10.1007/s00330-021-07803-2	Liver lesion detection
https://github.com/pjreddie/darknet	10.3348/kjr.2020.0447	Liver lesion detection
https://github.com/MIC-DKFZ/nnUNet	https://doi.org/10.3390/cancers13112726	Liver lesion segmentation
https://github.com/OnofreyLab/liver-segm/	https://doi.org/10.1371/journal.pone.0260630	Liver segmentation
Code in supplementary files	https://doi.org/10.1038/s41416-021-01511-w	Liver lesion detection
https://github.com/ETVP/Multi-Modal-Co-learning	10.1109/TMI.2021.3089702	Liver lesion segmentation
https://github.com/shuchao1212/TA-Net	https://doi.org/10.1016/j.neunet.2021.03.006	Liver lesion segmentation
https://github.com/linhandev/medSeg	https://doi.org/10.1016/j.compeleceng.2021.107118	Liver segmentation
https://github.com/rsummers11/CADLab/tree/master/CT%20Liver%20Segmentation%20Software	https://doi.org/10.1148/radiol.2021210531	Liver segmentation
https://github.com/seungsoolee0007/liver_spleen_segmentation	https://doi.org/10.3390/diagnostics12030590	Liver segmentation and right-libe graft weight estimation
https://github.com/neemajamshidi/PADLLS	https://doi.org/10.1038/s41598-022-20108-8	Liver segmentation
http://github.com/AdarLux/LiverUS.git	https://doi.org/10.1007/s00423-022-02674-7	Liver lesion detection
https://github.com/zfy012/Ite-netpp	https://doi.org/10.1080/08839514.2022.2151186	Liver segmentation
https://github.com/hicccp/liver-and-tumor-auto-segmentation.	https://doi.org/10.1186/s12885-023-11432-x	Liver lesion segmentation
https://github.com/wushu526/small_liver_tumor_segmentation	https://doi.org/10.3390/diagnostics13152504	Liver lesion detection and segmentation
https://github.com/FujifilmMedicalSystemsJapan/LiverSegmentation	https://doi.org/10.1002/jhbp.1357	Liver segmentation

https://github.com/frtozcan/trakyauniv	https://doi.org/10.3390/bioengineering10020215	Liver parenchyma and lesion segmentation
https://git.uibk.ac.at/informatik/igs/open/zigpub	https://doi.org/10.1007/s11548-023-02912-3	Liver segmentation
https://github.com/lzhLab/LiVS	10.1109/TMI.2023.3273528	Liver vessel segmentation
https://github.com/VesperCi/Capsule-Network-for-Liver-CT-Images	https://doi.org/10.3390/info14030183	Liver detection
https://github.com/lzhLab/LSM	10.21037/qims-22-1399	Liver lesion segmentation
https://github.com/OnofreyLab/volumetry-net	https://doi.org/10.1007/s00330-023-10495-5	Liver segmentation
https://github.com/huaxiangliu/S2DA-Net	https://doi.org/10.1016/j.compbiomed.2024.108400	Liver lesion segmentation
https://github.com/killian-zero/py_tumor-segmentation.git	https://doi.org/10.1177/15330338231219366	Liver lesion segmentation
https://github.com/Jouiry/ResTransUNet	https://doi.org/10.1016/j.compbiomed.2024.108625	Liver segmentation
https://github.com/alibool/detect-then-track	https://doi.org/10.1007/s10278-024-01132-8	Liver lesion detection
https://github.com/lzhLab/cross-modal-guidance	https://doi.org/10.1016/j.bspc.2023.105561	Liver lesion segmentation
https://github.com/mist-medical/MIST https://pypi.org/project/mist-medical/	https://doi.org/10.1038/s41598-024-71674-y	Liver segmentation
https://github.com/lqanh11/Interactive_AblationZone_Segmentation	https://doi.org/10.1002/mp.17373	Ablation zone segmentation
https://github.com/lzhLab/veSeg/	https://doi.org/10.1186/s12880-024-01309-1	Liver vessel segmentation
gitlab.in2p3.fr/iftim/public-projects/navigating-the-nuances	https://doi.org/10.1038/s41598-024-53528-9	Liver parenchyma and lesion segmentation
https://github.com/NewOneNow/EG-UNETR	https://doi.org/10.1016/j.bspc.2024.106739	Liver lesion segmentation
https://github.com/zephyrize/TAGNet	https://doi.org/10.1016/j.bspc.2023.105244	Bile ducts segmentation
